# Supplementary material for: Parental/Guardian–Child Physical Activity in Relation to Racial/Ethnic Inequities in the Americas: A Scoping Review
Source: Healthcare (Basel). 2025 Dec 1;13(23):3130. doi: 10.3390/healthcare13233130 (PMC12691846; doi:10.3390/healthcare13233130)

Supplementary Table S1. Descriptive information relating to the PAGER methodology of the scoping review parental/Guardian–Child PA in Relation to Racial/Ethnic Inequities in the Americas.

PAGER Element	Description
Patterns	Across the included studies, a consistent positive association was observed between parental/guardian and child PA. Most studies have investigated physical activities during leisure time. But studies were conducted in the USA, only a small subset of studies (n = 7) provided indirect racial/ethnic comparisons and low analytical depth in the literature.
Advances	Recent studies have advanced the field by incorporating sociocultural and structural factors into intergenerational PA research and by using objective measures (e.g., accelerometers). The inclusion of race/ethnicity as an analytic variable marks an important methodological step forward, though still inconsistently applied. Although few in number, there are longitudinal studies and clinical trials that investigate the practice of intergenerational PA, providing evidence for these populations.
Gaps	Major gaps include the scarcity of studies conducting racial/ethnic comparisons, the absence of research outside the USA, particularly in Latin America and the Caribbean, and the lack of longitudinal or theoretically grounded approaches. Few studies have explored the influence of systemic racism, historical inequities, or structural determinants on PA behaviors within families. There is a heterogeneity in the instruments used to measure PA, limiting the comparison of results. However, the limited number of comparative analyses prevents definitive conclusions about racial and ethnic inequities. Rather than confirming inequities, the findings highlight a significant research gap, the underrepresentation of racial and ethnic dimensions in studies on intergenerational PA. Although searches were conducted in languages such as Portuguese and Spanish, in the databases that allowed for such searches, no articles were found other than in English.
Evidence for Practice	Despite limited comparative evidence, the findings suggest that family- and community-based PA programs should account for structural and cultural barriers affecting racialized families. Promoting intergenerational and culturally tailored strategies could enhance equity in PA participation and health outcomes.
Research Recommendations	Future research should (1) systematically include race/ethnicity variables and conduct stratified analyses; (2) expand geographic scope beyond the USA.; (3) reanalyze existing datasets for racial/ethnic inequities; (4) integrate critical and intersectional theoretical frameworks; and (5) investigate how structural determinants (e.g., racism, socioeconomic inequalities, policy environments) shape intergenerational PA.

USA: United States of America; PA: Physical Activity.

Supplementary Table S2. General information on studies investigating parental/guardian and child PA and race and ethnic background (n = 25).

Authors and Year	Objective of the study	Methodological details	Main results
Alhassan et al., 2018	To examine the feasibility and efficacy of a 12-week culturally-tailored mother-daughter PA intervention on the PA levels of pre-adolescent African-American girls.	This study was a three-arm, parallel group, pilot randomized controlled trial. Dance intervention time PA: In the effect of experimental group (child alone and child-mother) on rates of PA intensities percent change over time with age and sexual maturation as covariates in the model.	The child-mother group had a significantly steeper rate of increase in percent time spent in vigorous PA (VPA) during the intervention period (γ = 1.05, p = 0.002), and in percent time spent in MVPA during the intervention period compared to child alone daughters (γ = 1.57, p = 0.007). Daughters in the CH-M group displayed significantly steeper rates of increase over time in percent time spent in VPA compared to the CON group ($\gamma = 0.80$, $p < 0.001$) and the CH group ($\chi 2$ (1) = 13.01, $p < 0.001$). Daughters in the CH group showed a significantly steeper rate of decrease in percent time spent in MVPA compared to the CON group ($\gamma = -1.29$, $p = 0.002$) and the CH-M group ($\chi 2$ (1) =26.40, $p < 0.001$), while the CH-M and CON group slopes revealed no significant differences.
Cason-Wilkerson et al., 2015	To understand how low-income, predominantly Hispanic, families accepted a family-based, lifestyle-change intervention and how they integrated treatment goals into daily life.	Focus groups were conducted with parents who participated in the Healthy Living Program, a community-based, family-oriented childhood obesity treatment program serving low-income, predominantly Hispanic, families in metropolitan area.	They made positive PA by learning specific skills and including the whole family in those changes. After completing the program, participants reported being more frequently active as a family through walks to the park, roller skating, dancing, parking farther away from stores to increase walking distance, climbing stairs,

			and enrolling children in organized sports.
Eisenberg et al., 2014	To explore whether recommendations for activity promotion are equally relevant to different adolescent populations.	Data come from two linked cross-sectional, population-based studies, EAT 2010 (Eating and Activity in Teens) and Families and Eating and Activity in Teens, addressing BMI, dietary intake, activity, and related factors among adolescents. Included 2,374 adolescents and their parent(s), recruited through 20 public schools in Minneapolis/St.Paul, in 2009–2010.Interactions of ethnicity/race and family/home environment.	Positive association between Adolescent MVPA and Parent's weekly hours of MVPA (β=.09, $p<.001$).
Garcia et al., 2021	To explore grandparent involvement in the care of Latino children in South Texas and their possible influence on child body mass index and family health behaviors to determine if they present an opportunity to improve child obesity treatment plans.	Were conducted an exploratory descriptive study in a 750-student elementary school, composed of approximately 90% Hispanic/Latino students, located in low-income areas in South Texas. Partnering with the local school district, we surveyed parents (N = 174) and grandparents (N = 108) of 188 Latino, primary school-age children regarding grandparent care and family health behaviors. We weighed, measured, and interviewed children regarding grandparent care and their own health behaviors.	Did find moderately strong significant relationships between times/week the child exercised and times/week the parent exercised ($r = .33$, $p = .001$, $n = 100$) as well as times/week the grandparent exercised ($r = .25$, $p = .012$, $n = 99$).
Jago et al., 2004	To obtain information about the relationship between cardiovascular and diabetic risk factors of mothers and their children and to determine whether these relationships differ by PA or ethnicity	Maternal PA and child PA was assessed by heart rate monitoring. Correlational methods were used to describe the relationships among metabolic risk factors and PA.	There was no significant (P.05) association between the physical activity levels of mothers and children.
Jang et al., 2016	To examine child, family and environmental factors associated with BMI in school-aged Korean American children. And examined the association of family and environmental factors	Convenience sampling methods with multiple strategies were used to recruit participants from Korean churches, Korean language schools and Korean restaurants and grocery stores in northeastern areas in the USA. A	There was no significant relationship between family or societal/demographic/community characteristics and child VPA.

	on child health behaviors in Korean American families.	Korean American family was defined as a biological family with a self-identified Korean or Korean American parent(s) and their children living in the USA.	
Nichols-English et al., 2006	To examine the association between Black mothers' and daughters' body fatness, PA beliefs and levels of PA, and are these variables predictive of daughters' PA behavior.	Black girls recruited from elementary schools in a low socioeconomic area. Data collected at the Georgia Prevention Institute.	No correlation between mothers' and daughters' moderate PA behavior (rs = 0.05). Mothers' and daughters' vigorous PA behavior was inversely associated (Rs = -0.16).
Pangalangan; Puma, 2024	To promote healthy behaviours, health promotion interventions must consider the entire family unit. This study aimed to assess the impact of parent's health behaviors on children's health behaviors in a sample of non-Hispanic and Hispanic low-income parents.	The study was a part of the Culture of Wellness in Preschools Programme, a multi-level obesity prevention programme. The data were obtained from the parent wellness workshops; a direct education intervention aimed at increasing fruit and vegetable consumption and PA among parents and their children.	Although still significant parent PA predicted very little of the variance in child PA levels ($R2 = .12$, $p < 0.001$).
Polley et al., 2005	To assess the prevalence of overweight in African American and Native American families and also to examine correlates of childhood obesity in families. Ethnic groups were compared because overweight and diabetes are more prevalent in Native Americans than in African Americans in Oklahoma.	A convenience sample of 84 three-generation families was recruited from 10 sites in rural Oklahoma. Families were recruited through community contacts at health, senior, community, and tribal centers.	There was no significant relationship between child PA and parent activity level.
Porter, 2017	To determine differences in blood pressure, dietary patterns, and PA practices among African Americans, Filipino Americans, and Hispanic Americans in South Florida; and to determine differences in dietary patterns and PA practices among fathers, mothers, and children.	The Background Information Questionnaire was used to gather information on age, gender, weight, race/ethnicity, family composition, and grade level of parents and children, as well as mothers' blood pressure	Mothers spent significantly more leisure time than their children; however, leisure time classes (including exercise classes) were attended more frequently by children, averaging 2 to 2.1 hour per week.
Ruiz et al., 2011	To examine the extent to which Hispanic parents' PA levels correlate with their preschool-aged child's PA patterns.	Was conducted at a local community center in Nashville, Davidson County, Tennessee. The PA was obtained from ActiGraph GT1M accelerometer	There were statistically significant mean differences between parent and child and PA levels such that parents were less physically active than their preschool-

		data collected as raw activity counts without imposition of any external criteria other than determination of wear and no wear time assessed using	aged child. Parent-child Pearson correlations in activity level were significant and large for low (r = 0.895; p < .0001) and moderate (r = 0.739; P < .0001) PA levels but not for vigorous PA levels (r = 0.07; P = 0.56). Parent-child correlation in overall PA was Pearson r = 0.61 (Spearman r = 0.58).
Sallis et al., 1988	To report familial aggregation of PA was studied using standardized and validated measure. In addition, aggregation was assessed in both Anglo and Mexican-American families.	The data reported here were gathered as part of a trial of family-based cardiovascular health education known as the San Diego Family Health.<br>PA habits were assessed by standardized interview in adults and children in 95 Anglo families and 111 Mexican-American families.	For the measure of hard leisure activity, the mother's activity was significantly correlated with scores for both children, although the correlation was much higher for older children. For Mexican-Americans the measure of hard leisure activity, the mother's activity was significantly correlated with scores for both children, although the correlation was much higher for older children. Family aggregation of vigorous leisure activity was less pronounced. For this measure the mother's exercise was related to both younger and older children.
Salvo et al., 2019	To identify child-, parental-, familial-, community-, and neighborhood-level factors associated with differences in 4-year changes in BMI, healthy eating, and PA, among children residing in low-income, predominantly Hispanic urban enclavesin Austin, Texas.	This exploratory secondary data analysis used baseline and follow-up cohort data from the Go Austin! Vamos Austin! Evaluation Evaluation Study, obtained from residents of selected zip codes located in Austin's Eastern Crescent.	After adjusting for covariates, marginal associations between having physically active parents (OR: 0.80, 95% CI: 0.39, 1.02) with children being in the "decreasing PA" group were observed.
Sweeney et al., 2023	To address this gap by evaluating a multitheoretical approach to engaging African American families in a tailored web-based weight loss program, including a novel focus on positive parenting practices.	Participants were recruited through community partnerships, culturally relevant advertisements, and community events.<br>Parent-adolescent dyads were randomized to a web-based tailored intervention or web-based health education	The results also indicated that engagement with different behavioral content sessions was not significantly related to MVPA after the web-based intervention among adolescents, suggesting that the effects observed in

		comparison program. The web-based intervention (N = 119) was completed by parents and targeted 6 weight-related behaviors to support their adolescent children's weight loss goals (session contents included energy balance, fast food, fruits and vegetables, PA, sedentary behavior, and sweetened beverages). MVPA was measured using accelerometers at baseline and after the intervention.	parents did not carry over to their adolescent children.
Trost et al., 1999	To identify the psychosocial and environmental correlates of PA behavior in a diverse sample of sixth-grade children.	Subjects for this study were 213 sixth-grade students from 4 public middle schools in Columbia, South Carolina. The study group was 51.6% female, 55.9% African-American, with a mean age of 11.4 +- 0.6 years	For boys, PA, perception of mother's activity level, were significant correlates of VPA. For girls, there was no association.
Wen; Su, 2015	To collect information on family demographics, socioeconomic status, health insurance coverage, diet, PA, family history of diabetes, and other variables for AN-positive students identified in the Texas Risk Assessment for Type 2 Diabetes in Children program.	Data used in this study were from the 2012 Survey on Family Background Associated with Acanthosis Nigricans.	Parental time spent in doing housework (OR = .76, p = .03) are negatively associated with child LPA participation levels whereas parental PE participation (OR = 2.20, p < .01) is a positive correlate.
Wilson et al., 2022	To test the efficacy of a cultural tailoring, positive parenting, and motivational intervention for weight loss in overweight African American adolescents.	Families were recruited through community partnerships such as local churches, pediatric clinics, schools, community events, and through culturally relevant ads. The trial tested an 8-week face-to-face group motivational plus family weight loss program compared with a comprehensive health education control program. Participants were then rerandomized to an 8-week tailored or control online program to test the added effects of the online intervention on reducing body mass index and improving MVPA, light PA, and diet.	At 16 weeks, there was a significant interaction between the group intervention and time for parent LPA (B = 33.017, SE = 13.115, p = .012, d = 0.671).

Wirthlin et al., 2020	To examine associations between parent modelling of PA and dietary intake and children's PA and dietary intake in a diverse sample residing in a metropolitan area in the USA.	This study is a secondary data analysis of data from the Family Matters study, a mixed-methods study examining risk and protective factors for childhood obesity in the home environment of racially/ethnically diverse families.	No significant association was observed between parent modelling of physical activity and child time MVPA (p = 0·14). When parent's light physical activity (e.g., walking, leisure sports like playing catch or table tennis and light yard/housework) increased, child light physical activity also increased.
Duncan; Strycker; Chaumeton, 2015	To examine associations between personal, family, and peer factors with MVPA and sports participation of African American, Latino, and white girls.	Data are from the first year of a study of 372 African American (n = 128), Latino (n = 120), and white (n = 124) girls residing in a Northwestern USA metropolitan area. Families having a 10-, 12-, or 14-year-old girl were randomly recruited using telephone, door-to-door, and word-of-mouth methods.	This model also revealed significant differences across ethnic groups for the mean of the sports participation latent factor (African American girls significantly higher than Latino and white girls). African American girls had a significantly higher mean for the sports participation latent factor and MVPA, and a significantly lower mean for friends' PA. White girls had the highest PA barriers efficacy and home exercise environment mean. The means for parental PA were significantly lower for Latino girls. White families had a significantly higher mean income than the other two groups (African American and Latino).
Gottlieb; Chen, 1985	To study the cultural patterns of exercise that might relate to future risk for heart disease.	Relative contribution of sex, ethnicity, social class, parental exercise and heart health knowledge of the variability of sporting activities reported by Texas 7th and 8th grade students in 1980 was examined to study the cultural patterns of exercise that might relate to future risk for heart disease. Girls were more likely than boys	Sex was the variable most strongly related to exercise frequency, followed by parental exercise. Father's occupation had the smallest relationship to exercise frequency. Examination of the interaction between sex and parental exercise on the students' exercise frequency showed that girls' exercise frequency increased more

		to participate in activities with high aerobic potential.	than that of boys as the number of parents exercising increased from 0 to 2. Parents should be encouraged to provide role models for their children’s health behaviors.
Madsen; McCulloch; Crawford, 2009	To determine whether parent modeling predicted girls’ activity, to determine whether the association declined with time, and to identify variations in this association by race.	Longitudinal examination of parent modeling’s impact on future log transformed metabolic equivalents (log METs) of LPA in 1213 African-American and 1166 Caucasian girls in the National Heart, Lung, and Blood Institute Growth and Health Study, from age 9 to 10 years through 18 to 19 years, using linear regression. Race interaction terms and time trends were examined.	Race and parent education explained 25% to 75% of the decrease in magnitude for mother’s and father’s activity, but none of the decrease for exercising with parent (although African American girls reported significantly lower levels of parental PA than Caucasian girls in years 3-8, they reported higher rates of exercising with a parent in years 7 and 8—data not shown).
McMurray et al., 2016	To compare the PA and sedentary time of obese, low-income, ethnic minority parents and their children on weekdays and weekend days using accelerometry.	The purpose of this study was to compare the PA and sedentary time of obese, low-income, ethnic minority parents and their children on weekdays and weekend days using accelerometry.	Mother-daughter correlations for reported PA and for beliefs and attitudes about exercise revealed low-order, generally nonsignificant correlations; only the question "I play sports or very active games a lot" was correlated significantly, and this occurred only in White mother-daughter pairs (r = .10, p = .007).
Morrison et al., 1994	To evaluate obesity as a potential explanatory factor for the increased relative risk for cardiovascular disease in black compared to white women.	Briefly, it is a cohort study of the development of obesity in Black and White adolescent females and of the effects of obesity on cardiovascular disease risk factors.	Mother-daughter correlations for reported PA and for beliefs and attitudes about exercise revealed low-order, generally no significant correlations; only the question "I play sports or very active games a lot" was correlated significantly, and this occurred only in White mother-daughter pairs (r = .10, p = .007).
Tandon; Zhou; Christakis, 2012	To quantify preschool-aged children’s parent- supervised outdoor play frequency and characterize children who are	Cross-sectional using data from the Early Child-hood Longitudinal Study–Birth	Mother’s race/ethnicity (OR for Asian, 0.51, 95% CI, 0.43-0.61; Black: 0.59, 95% CI, 0.49-0.70; Hispanic: 0.80,

	most at risk for less frequent parent-supervised outdoor time.	Cohort. Nationally representative USA sample. Participants: Preschool-aged children. Main Outcome Measure: Parent-reported outdoor play frequency.	95% CI, 0.67-0.95), mother's employment (OR for full time, 0.70; 95% CI: 0.62-0.81), and parent's exercise frequency of 4 days or more per week (OR, 1.50; 95% CI, 1.28-1.75).
Trost et al., 1997	To understanding the factors that influence PA behavior is important in the design of intervention programs targeted at youth.	A prospective study design was used to identify the predictors of vigorous PA (VPA) (> 6 METs) and moderate and vigorous PA (MVPA) (> 3 METs) among 202 rural, predominantly African-American children. Selected social cognitive determinants of PA were assessed via questionnaire in the fifth grade. Participation in VPA and MVPA was assessed via the previous day PA recall 1 year later in the sixth grade.	For girls, participation in community sports teams, self-efficacy in overcoming barriers, enjoyment of physical education, race/ethnicity, and perception of mother's activity entered the regression model at the $p < .05$ level, accounting for 26% of the variance in VPA. race/ethnicity (white vs African American), and enjoyment of school physical education were significant correlates of VPA. For girls, self-efficacy (overcoming barriers), community sports teams, race/ethnicity, and enjoyment of school physical education were significant correlates of MVPA.

PA: physical activity; RN: not reported; MPA: physical activity moderate; VPA: vigorous physical activity, MVPA: moderate to vigorous physical activity, LPA: physical activity leisure, METs: Metabolic Equivalent of the Task, OR: odds ratio, USA: United States of America; p = p-value (probability value); Y = indicates that the study reported an association.; BMI: body mass index; IC: confidence interval. CH-M: child-mother; CON group: control; CH: child alone.

Supplementary Table S3. Characterization information of the study samples composed of parental/Guardian–Child in each of the studies in the review.

Authors and Year	Number of Dyads	Sample of parents/guardians	Sample of child	Average Age of Parents	Average Age of Child	Parents/Caregivers	Sex Child/Adolescent	Adolescent or Child
Studies that associated the practice of PA between parents and children (18 studies)								
Alhassan et al., 2018	32	32	32	37.4 SD 7.7	9 ± 1.4 years	Mother	Female	Child
Cason-Wilkerson et al., 2015	NR	NR	37	NR	8 - 12	Mother and father	Both	Both
Eisenberg et al., 2014	NR	3.709	2.374	42.4 SD 8.4	14.4 SD 2.0	Mother and father	Both	Adolescent
Garcia et al., 2021	101	174 parents 108 Grandparents	188	33.3 SD 7.9	8.3 SD 1.8	Mother, father and grandfather.	Both	Child
Jago et al., 2004	133	133	133	32.1 SD 5.6	6.5 SD 0.6	Mother	Both	Child
Jang, 2016	NR	170	137	42.7 SD 3.9	10.9 SD 2.0	Mother	Both	Child
Nichols-English et al., 2006	133	133	133	37.1 SD 7.8	9.6 SD 0.8	Mother	Female	Both
Pangalangan; Puma, 2024	NR	410	NR	>18	1-11 years	Mother and father	Both	Child
Polley et al., 2005	84	87	87	NR	NR	Other	Both	Child
Porter, 2017	NR	NR	110	22.56-13.53-12.67	NR	Mother and father	Both	Both

Ruiz et al., 2011	80	85	80	31.16 SD 5.5	4.2 SD 0.9	Mother and father	Both	Child
Sallis et al., 1988	206	289	247	Anglo Fathers: 39.1 SD 5.9 Mothers: 37.2 SD 5.3 Mexican-American Fathers: 37.9 SD 6.9 Mothers: 35.4 SD 6.0	Anglo: 11.4 SD 1.3 Older: 13.2 SD 2.5 Mexican-American Younger: 11.6 SD 0.9 Older: 12.5 SD 2.2	Mother and father	Both	Both
Salvo et al., 2019	286	286	286	>18 years	6 years	Mother and father	Both	Child
Sweeney et al., 2023	119	119	119	43.69 SD 8.94	12.71 SD 1.67	Mother and father	Both	Both
Trost et al., 1999	NR	NR	102	NR	11.4 SD 0.6	Mother and father	Both	Both
Wen; Su, 2015	305	305	305	NR	10.5	Mother and father	Both	Both
Wilson et al., 2022	241	241	241	GIOI :44.19 SD 9.40; GIOC: 42.49 SD 8.59; GCOI:43.21 SD 8.61;	GIOI: 12.81 SD1.73 GIOC: 13.00 SD 1.87; GCOI: 12.61 SD 1.63; GCOC: 12.9 SD 1.80.	Mother and father	Both	Adolescent

				GCOC: 42.89 SD 8.10.				
Wirthlin et al., 2020	150	150	150	34.5 SD 7.1	6.4 SD 0.8	Mother and father	Both	Child
Studies that revealed racial/ethnic inequities in the practice of PA between parents and children (7 studies)								
Duncan; Strycker; Chaumeton, 2015	372	372	372	NR	12.06 SD 1.69	Mother and father	Female	Adolescent
Gottlieb; Chen, 1985	NR	NR	NR	NR	11 - 15	Father	Both	Both
Madsen; McCulloch; Crawford, 2009	NR	NR	2739	NR	African American:10.1; Caucasian: 10.0	Mother and father	Female	Both
McMurray et al., 2016	199	199	199	37.5 SD 7.9	9.0 SD 0.9	Mother and father	Both	Child
Morrison et al., 1994	1300	White: 720 Black: 579	White: 720 Black: 580	White: 37.7 SD 5.2; Black: 35.7 SD 5.5.	White: 10 SD 0.6; Black: 10.1 SD 0.5.	Mother	Female	Child
Tandon; Zhou; Christakis, 2012	8950	8950	8950	32.16 SD 0.14	4.37 SD 0.01	Mother	Both	Child
Trost et al., 1997	NR	NR	202	NR	11 - 14	Mother and father	Both	Both

PA: physical activity; GIOI: Group intervention and online intervention; GIOC: Group intervention and online control; GCOI: Group control and online intervention, NR: Not related, SD: Standard Deviation

Supplementary Table S4. Information on measures of PA of parents/caregivers and children/adolescents in each of the studies in the review.

Authors and year	Domain	Weekly frequency	Duration/Time	Intensity	Measurement method	Information about the data collection instrument	Number of days	Questions/cutoff point
Studies that associated the practice of PA between parents and children (18 studies)								
Alhassan et al., 2018								
Parents /caregivers	_	Yes	Yes	Moderate; Vigorous	Accelerometer	Accelerometer (Actigraph, LLC, Pensacola, FL)	Seven days	Vigorous ≥5999 counts/min and MVPA ≥2020 counts/min
Children/adolescent	_	Yes	Yes	Moderate; Vigorous	Accelerometer	Accelerometer (Actigraph, LLC, Pensacola, FL)	Seven days	Vigorous ≥4012 counts/15s and MVPA ≥2296 counts/15s
Cason-Wilkerson et al., 2015								
Parents /caregivers	_	NR	NR	NR	Questionnaire	Qualitative	_	
Children/adolescent	_	NR	NR	NR	Questionnaire	Qualitative	_	
Eisenberg et al., 2014								
Parents /caregivers	Laisure	Yes	Yes	Moderate; Vigorous	Questionnaire	Leisure Time Exercise Questionnaire	_	"In a typical week, how many hours do you spend…Being physically active with your child (e.g., throwing a ball around, taking a walk or bike ride together)? Helping your child to be

								physically active (e.g., driving them to the gym or sport practice, watching them play a sport)?”
Children/adolescent	Laisure	Yes	Yes	Moderate; Vigorous	Questionnaire	Leisure Time Exercise Questionnaire	_	“In a typical week, how many hours do you spend…Being physically active with your child (e.g., throwing a ball around, taking a walk or bike ride together)? Helping your child to be physially active (e.g., driving them to the gym or sport practice, watching them play a sport)?”
				Garcia et al., 2021				
Parents /caregivers	_	Yes	NR	NR	Questionnaire	Question addressing frequency of strenuous PA	_	_

Children/adolescent		Yes	NR	NR	Questionnaire	Question addressing frequency of strenuous PA	_	_
Jago et al., 2004								
Parents /caregivers	_	Yes	Yes	Vigorous.	Questionnaire	Seven-Day PA Recall	Seven days	_
Children/adolescents	_	Yes	Yes	Vigorous.	Telemetry	Heart rate monitor (Quantum XL; AMF American, Jefferson, Iowa)	Seven days	Preprogrammed to record for the entire day was attached to each child's chest and removed by a technician at approximately 7 PM.
Jang, 2016								
Parents /caregivers	_	Yes	Yes	Vigorous.	Questionnaire	IPAQ-short	_	_
Children/adolescent	_	Yes	Yes	Vigorous.	Questionnaire	Subscale of the elementary-level School-based Nutrition Monitoring Questionnaire.	_	For physical activity, data were collected on 'vigorous PA for at least 30 minutes during last week' and 'sport team participation during the past 12 months'.
Nichols-English et al., 2006								
Parents /caregivers	_	Yes	Yes	Moderate; Vigorous	Questionnaire	Seven Day PA Recall and the 16-	Seven days	Average number of minutes of PA

						item Relative Fitness Value Scale		(moderate, hard, and very hard) per day that were performed in the past seven days
Children/adolescent	_	Yes	Yes	Moderate; Vigorous	Questionnaire	Seven Day PA Recall	_	_
Pangalangan; Puma, 2024								
Parents /caregivers	_	No	No	NR	Questionnaire	The 28-item Family Wellness Summary pre-survey. Parents were asked to answer these questions for both themselves, and their child	_	_
Children/adolescent	_	No	No	NR	Questionnaire	The 28-item Family Wellness Summary pre-survey.	_	_
Polley et al., 2005								
Parents /caregivers	_	Yes	Yes	Vigorous	Questionnaire	The questionnaire consisted of 32 questions on socioeconomic, health, diet, and PA information	_	_
Children/adolescent	_	Yes	Yes	Vigorous	Questionnaire	The questionnaire consisted of 32 questions on socioeconomic,	_	_

						health, diet, and PA information. Activity levels were self-reported and choices ranged from one (no regular exercise program) to six (more than 3 hours weekly or 30 minutes daily of vigorous physical activity)		
Porter, 2017								
Parents /caregivers	_	Yes	Yes	NR	Questionnaire	Family Eating and Activity Habits Questionnaire	_	_
Children/adolescent	_	Yes	Yes	NR	Questionnaire	Family Eating and Activity Habits Questionnaire	_	_
Ruiz et al., 2011								
Parents /caregivers	_	Yes	Yes	Moderate; Vigorous	Accelerometer	Accelerometer (Actigraph, LLC, Pensacola, FL)	7 consecutive days during waking hours except when they showered or participated in aquatic activities.	The thresh-olds were 500 counts for sedentary, 500 to 2019 counts for light, 2020 to 5999 counts for moderate, and 6000

								counts for vigorous activity counts per minute.
Children/adolescent		Yes	Yes	Moderate; Vigorous	Accelerometer	Accelerometer (Actigraph, LLC, Pensacola, FL)	7 consecutive days during waking hours except when they showered or participated in aquatic activities.	thresholds of 420 activity counts for sedentary, 420 to 1679 counts for light, 1680 to 3379 counts for moderate, and 3379 counts for vigorous intensity activity per minute.
Sallis et al., 1988								
Parents /caregivers	Work; Leisure	No	Yes	Moderate; High; Very High-intensity	Questionnaire	Seven day PA Recall	Seven days	_
Children/adolescent	No organized PA; Active work.	No	Yes	Moderate; High; Very High-intensity	Questionnaire	Seven day PA Recall	Seven days	_
Salvo et al., 2019								
Parents /caregivers	General	Yes	Yes	Moderate; Vigorous.	Questionnaire	Modified version of the self-	This variable	_

						administered Past week PA questionnaire	was dichotomized, based on the Physical Activity Guidelines for Americans (≥150 min per week, vs. <150 min per week).	
Children/adolescent	General	Yes	Yes	Moderate; Vigorous.	Questionnaire	"Reported by parents/caregivers by the question: "	_	How many days per week is your child physically active for at least 30 min?” For both items, response options included “less than 1 day per week,” “2–3 days per week,” “4–5 days per week,” and “more than 5 days per week,” and were scored to represent weekly frequency of these behaviors.

Sweeney et al., 2023								
Parents /caregivers	_	Yes	Yes	Moderate; Vigorous	Accelerometers	Accelerometer (Omnidirectional Actical)	7 consecutive days	MVPA (counts >1535)
Children/adolescent	_	Yes	Yes	Moderate; Vigorous	Accelerometers	Accelerometer (Omnidirectional Actical)	7 consecutive days	MVPA (counts >1500)
Trost et al., 1999								
Parents /caregivers	_	Yes	No	NR	Questionnaire	Perception of parent's activity level	–	–
Children/adolescent	_	Yes	Yes	Moderate; Vigorous	Accelerometer	Computer Science and applications Inc. (CSA) 7164 activity monitor. Uniaxial accelerometer (Shalimar, Florida).	Seven consecutive days	NR
Wen ; Su , 2015								
Parents /caregivers	Leisure; Domestic activities	Yes	No	NR	Questionnaire	Two questions	_	"On a typical day, how much time (in minutes) do you spend on physical exercises such as walking, jogging, hiking, swimming, or playing a ball game?". "On a typical day, how much

								time (in minutes) do you spend on housework such as cleaning, gardening, or cutting grass?” "
Children/adolescent	Organized PA	Yes	No	NR	Questionnaire.	One question	_	“On average, how often does your child have physical exercises or sports after school?”
Wilson et al., 2022								
Parents /caregivers	_	Yes	Yes	Moderate, Vigorous; Light.	Accelerometer	Accelerometer (Omnidirectional actical)	Seven consecutive days from omnidirection al	MVPA (counts above 1,535) and LPA (counts between 110 and 1,534
Children/adolescent	_	Yes	Yes	Moderate; Vigorous; Light	Accelerometer	Accelerometer (Omnidirectional actical)	Seven consecutive days from omnidirection al	MVPA (counts above 1,500) and LPA (counts be-tween 100 and 1,500
Wirthlin et al., 2020								
Parents /caregivers	_	Yes	Yes	Moderate; Vigorous.	Accelerometers	Accelerometers (ActiGraph LLC)	Seven consecutive days	_
Children/adolescent	_	Yes	Yes	Moderate; Vigorous	Accelerometers	Accelerometers (ActiGraph LLC)	Seven consecutive days	LPA: 101–2.295, MPA: 2.296–

								4.011, e VPA ≥4.012
Studies that revealed racial and ethnic inequities in the practice of PA between parents and children (7 studies)								
Duncan; Strycker; Chaumeton, 2015								
Parents /caregivers	_	Yes	No	NR	Questionnaire	Behavioral Risk Factor Surveillance System	_	"In a typical week, on how many days are you [other adults in your household] physically active for a total of at least 30 minutes per day?"
Children/adolescent	_	Yes	Yes	Moderate; Vigorous	Accelerometer.	Accelerometer (GT3X+ ActiGraph)	_	MVPA≥ 2000 to 2999, and vigorous activity as over 3000
Gottlieb; Chen, 1985								
Parents /caregivers	Leisure	Yes	No	NR	Questionnaire.	Research questionnaire	_	_
Children/adolescent	Organized PA.	Yes	No	NR	Questionnaire.	Research questionnaire	_	_
Madsen; McCulloch; Crawford, 2009								
Parents /caregivers	Leisure	_	_	_	Questionnaire	Two questions	_	Do you exercise 3 or more times a week? Do you play sports or active games frequently? Do

								you run, play ball, exercise or take long walks at least 3 times a week?
Children/adolescent	Organized PA; No organized PA	_	_	_	Questionnaire	Habitual Activity Questionnaire	_	_
McMurray et al., 2016								
Parents /caregivers	_	Yes	Yes	Moderate; Vigorous	Accelerometer	Accelerometer (Actical, Philips Respironics, Bend, Oregon, USA)	Four days	LPA 100-1535; MPA: 1535-3960; VPA: ≥3960
Children/adolescent	_	Yes	Yes	Moderate; Vigorous	Accelerometer	Accelerometer (Actical, Philips Respironics, Bend, Oregon, USA)	Four days	LPA:100-1600; MPA:1600-4700; VPA: ≥4700
Morrison et al., 1994								
Parents /caregivers	Leisure PA	Yes	Yes	NR	Questionnaire	One question	_	"I play sports or very active games a lot"
Children/adolescent	Organized PA	Yes	Yes	NR	Questionnaire	One question	_	"I play sports or very active games a lot"
Tandon; Zhou; Christakis, 2012								
Parents /caregivers	Leisure PA	Yes	Yes	NR	Questionnaire	One question	_	“In the past month, how often did you take child outside for a

								walk or to play in yard, a park, or a playground?”
Children/adolescent	No organized PA	Yes	Yes	Moderate; Vigorous	Questionnaire	Answered by responsible parents	_	_
Trost et al., 1997								
Parents /caregivers	_	Yes	Yes	Moderate; Vigorous	Questionnaire	Child's perception	_	NR
Children/adolescent	_	Yes	Yes	Moderate; Vigorous	Questionnaire;	Previous day PA recall	_	_

NR: Not reported; PA: physical activity; IPAQ-Short: international physical activity questionnaire-short; MPA: physical activity moderate; VPA: vigorous physical activity, MVPA: moderate to vigorous physical activity, LPA: physical activity leisure; USA: United States of America.

Supplementary Figure S1. Showing the reasons for exclusion from the studies.

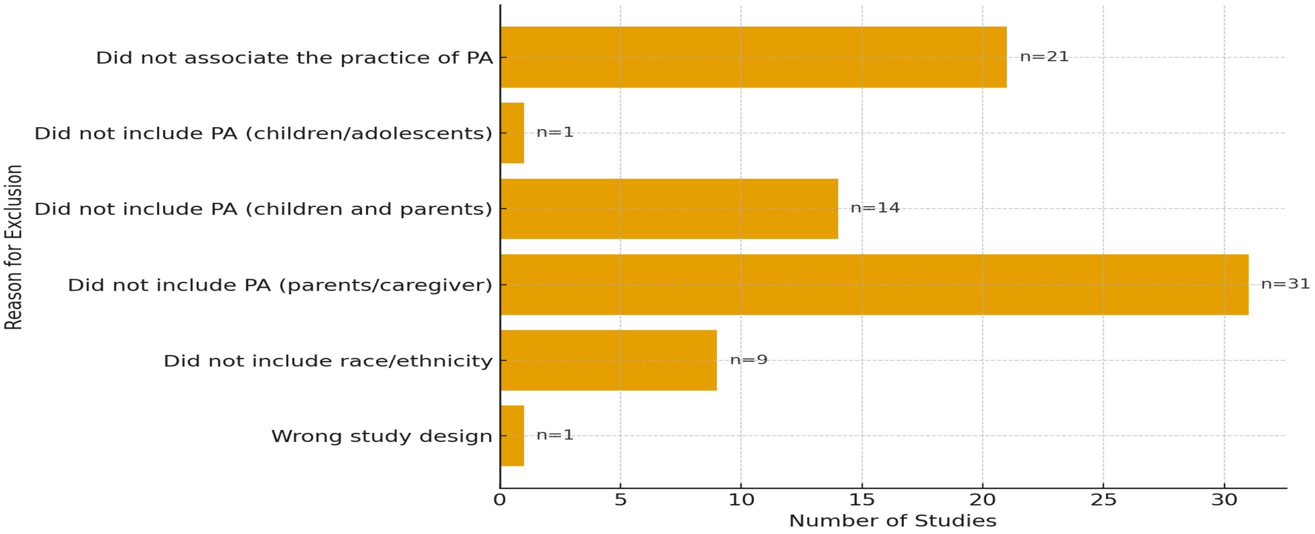

Supplement: Supplementary file 1 [file healthcare-13-03130-s001.zip › healthcare-3936165-supplementary.pdf]
